# Exciton-polariton Josephson junctions at finite temperatures

**DOI:** 10.1038/s41598-017-09824-8

**Published:** 2017-08-25

**Authors:** M. E. Lebedev, D. A. Dolinina, Kuo-Bin Hong, Tien-Chang Lu, A. V. Kavokin, A. P. Alodjants

**Affiliations:** 1 ITMO University, St. Petersburg, 197101 Russia; 2 Department of Photonics, National Chiao Tung University, Hsinchu, 300 Taiwan; 3 Spin Optics Laboratory, St. Petersburg State University, Ul’anovskaya, Peterhof, St. Petersburg 198504 Russia; 4 School of Physics and Astronomy, University of Southampton, SO17 1BJ Southampton, United Kingdom; 5 Istituto CNR-SPIN, Viale del Politecnico 1, I-00133 Rome, Italy; 6 Vladimir State University named after A. G. and N. G. Stoletovs, Gorkii Street 87, Vladimir, Russia

## Abstract

We consider finite temperature effects in a non-standard Bose-Hubbard model for an exciton- polariton Josephson junction (JJ) that is characterised by complicated potential energy landscapes (PEL) consisting of sets of barriers and wells. We show that the transition between thermal activation (classical) and tunneling (quantum) regimes exhibits universal features of the first and second order phase transition (PT) depending on the PEL for two polariton condensates that might be described as transition from the thermal to the quantum annealing regime. In the presence of dissipation the relative phase of two condensates exhibits non-equilibrium PT from the quantum regime characterized by efficient tunneling of polaritons to the regime of permanent Josephson or Rabi oscillations, where the tunneling is suppressed, respectively. This analysis paves the way for the application of coupled polariton condensates for the realisation of a quantum annealing algorithm in presently experimentally accessible semiconductor microcavities possessing high (10^5^ and more) Q-factors.

## Introduction

In the XXI century, the studies of exciton-polariton Bose-Einstein condensates (BEC) in various type of semiconductor microstructures have become an important area of research in photonics and semiconductor physics^1, 2^. Microcavity exciton polaritons are quasiparticles representing admixtures of quantized cavity photons and quantum well excitons. Semiconductor microcavities are promising for various optoelectronic applications where the quantum matter-field interface plays an essential role. In in such systems low branch polaritons can be treated as a 2D weakly interacting bosonic gas.

The polariton lasers are currently available optoelectronic devices for which coherent emission (“lasing”) occurs due to BEC of low branch exciton-polaritons. Bosonic condensates of exciton-polaritons have been observed at elevated temperatures ranging from a few tens of Kelvin in GaAs and CdTe based microcavities to the room temperature in GaN, ZnO and organic microcavities. Polariton lasing is a spectacular example of the formation of a bosonic condensate of exciton-polaritons in a driven-dissipative system. Among the recent achievements of polaritonics it is worth to mention experimental demonstrations of polariton lasers with electrical injection^3, 4^, polariton amplifiers^5^, switches^6^, transistors^7^, polariton circuits and optical logic elements^8, 9^. Although exciton-polariton condensates exhibit the Bose-Einstein statistics above the lasing threshold and are characterized by a macroscopic occupation of the ground state at certain pumping rate that is less than the threshold pumping for convenient lasers, they are not in a true thermodynamic equilibrium state^10, 11^. Non-equilibrium features of exciton polariton condensates play an important role in various manifestations of their collective (many body) states such as superfluidity^12, 13^, quantized vortices^14, 15^, soliton formation^16^, Josephson oscillations and macroscopic self-trapping^17, 18^.

The problem of distinguishability of statistically classical (thermal) and quantum regimes for exciton- polariton condensates is very important in view of the possible exciton-polaritons applications in quantum information technologies^19^. Fast switching properties (the typical switching time of a few picoseconds), relatively strong nonlinear response and flexibility to external optical and/or electrical pumping spin degrees of freedom made microcavity polaritons potentially promising for quantum computation and quantum information processing^19–23^.

**Figure 1:**
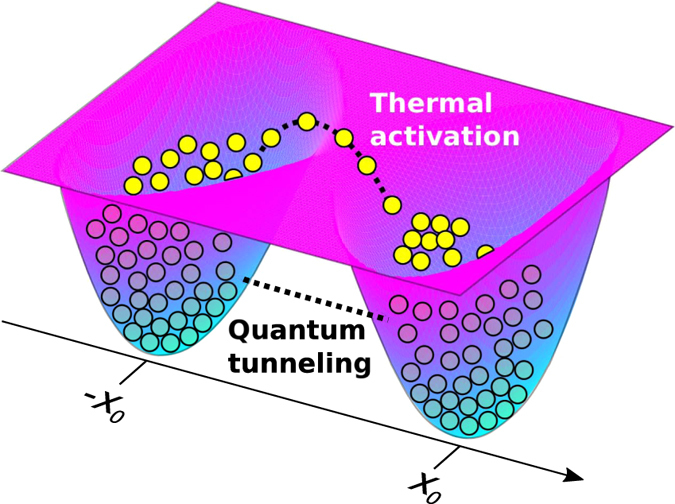
Sketch of two tunnel coupled exciton-polariton condensates. The two minima of the trapping potential *U* are situated at the points +*x*_0_ and −*x*_0_. At thermal equilibrium the interplay between thermal and quantum annealing effects is governed by the blue shift of condensates, effective temperature of exciton-polaritons and the shape of the potential.

Here we specifically focus on the quantum annealing problem that is relevant to the searching algorithm for the global minimum of the potential energy landscape (PEL) consisting of a set of barriers and wells^24, 25^. In a purely classical (thermal) regime the bosonic quasipartcles cross barriers stochastically at finite temperature with the help of thermal activation process if the thermal energy is large enough, see Fig. 1. Contrary, in a quantum limit the same system undergoes quantum tunneling through the barrier. Obviously, the shape (height and thickness) of the barrier plays an essential role in this case^25, 26^. It has been shown recently in ref. 26 that the annealing algorithm relies in general case on the combination of thermal annealing and quantum tunneling. The collective (bosonic) character of polariton condensation could be used for the acceleration of the physical implementation of the search algorithm^27^. The authors of refs 28, 29. propose polariton graphs as an analog platform for minimizing the XY–Hamiltonian by exploring the non-equilibrium character of exciton-polariton condensates and mimicking various magnetic phases with them. An original technique of nonresonant spatially modulated pumping beam have been used to imprint two-dimensional polariton graphs with different topology and different heights of potential barriers. Importantly, the phase-locking between arbitrary neighboring vertices might be practically achieved in this case.

In the present paper we study the effect of a finite effective temperature on the coupling of quasi-equilibrium exciton-polariton condensates accounting for the competing thermal and quantum annealing effects. To be more specific, here we examine an exciton-polariton Josephson junction (JJ) as a toy model for the classical or quantum annealing problem.

The Josephson effect initially discovered in superconductors^30^ then has been studied in weakly coupled atomic BEC’s^31–35^. Since the temperature of atomic Bose-Einstein condensation is extremely low (*μ*K’s and below) it is usually possible to consider dynamical effects occurring with two atomic condensates confined in a W-shape potential using the mean field approach in zero temperature approximation. Josephson and Rabi oscillations, self-strapping of atoms and the population imbalance between potential traps have been reported^36, 37^.

JJ of exciton-polariton condensates is a relatively young Josephson coupled system that is being studied in many experimental laboratories around the Globe^17, 18, 38–43^. It is important to underline that the polariton system is strongly different from superconducting and atomic JJs for several reasons, namely:

(i) In superconducting JJ the tunnelling of electrically charged Cooper pairs is at the origin of coupling, while in the case of exciton-polariton condensates, electrically neutral exciton-polaritons tunnel.
(ii) The mechanisms of dissipation are dramatically different in the systems. While Cooper pairs (or atomic BEC’s) are stable below critical temperature, exciton-polaritons are characterised by a radiative life-time: the features of the junctions essentially depend on the Q-factor of the cavity, which is why the exciton-polariton system needs to be considered as a driven-dissipative system by its origin
(iii) All the most essential parameters of exciton-polariton JJ and superconductor/atomic systems: masses, characteristic length- and time-scales are dramatically different, see refs 12, 40–42.

Recently Yongbao Sun *et al*. reported^44^ the observation of a quasi-equilibrium low branch exciton polariton condensate in a high Q-factor microcavity characterised by a remarkably long photon lifetime of 135 *ps*. Clearly, exciton-polaritons with long lifetime are promising candidates especially for quantum technologies^20^.

One of the advantages of exciton-polaritons over cold atoms consists in a perspective of room temperature operation of polariton devices. In this context, it is very important to reveal the impact of temperature on the physics of interacting polariton condensates. It should be also noted that the effective temperature of a polariton gas may be introduced in the quasi-equilibrium approximation, which is not necessarily valid in all cases but constitutes an important starting point of any analysis. Further steps would involve a full kinetic modeling for a non-equilibrium polariton gas. We study the interplay of two mechanisms of coupling between the condensates in order to reveal the cross-over from the quantum tunneling to the incoherent coupling regime that may occur at finite temperatures^38, 40, 43^. In particular, we are interested in a generic problem of the quantum-classical phase transition (PT) applied to macroscopic two-level systems^45–49^.

Traditionally, studies in this field are limited by consideration of superconductor devices^50^ where macroscopic quantum tunneling phenomena plays an essential role. In particular, it is worth to mention here the Schrödinger cat state formation^51^ and the design of quantum gates with superconductor qubits for quantum computing where a macroscopic quantum coherence for the phase is important^52^.

In this manuscript we specifically consider a couple of trapped exciton-polariton condensates in a high-Q GaAs microcavity. We take into account the driven-dissipative nature of the system, as well as the high temperature of the exciton-polariton gas. We go beyond the mean-field approximation adopted in the most part of theoretical works on exciton-polariton quantum fluids, and present the full phase diagram of the system. We shall consider a generalised model of exciton-polariton JJ that relates to so-called non-standard Bose-Hubbard models (see e.g refs 33, 53.) where the energy of polariton-polariton scattering contributes to the tunneling parameter matrix elements^38–41, 43^. In order, we pay attention to the temperature dependent quantum critical phenomena occurring in the presence of macroscopic tunneling. We aim at formulation of the criteria for realisation of the quantum tunneling regime that is important for the realisation of the quantum annealing algorithms and exciton-polariton quantum gates. The influence of non-equilibrium effects involving the microcavity exciton polaritons possessing a finite lifetime is discussed as well.

## The Quantum Phase Model

We consider a system of two spinless exciton-polariton condensates localised in lateral potential traps created in a planar semiconductor microcavity. The condensates are confined by a W-shape potential *U*(*x*) possessing two minima at the points ±*x*_0_, see Fig. 1. Polaritons are pumped to the condensates from a reservoir of incoherent excitons. In this and next Section, we shall neglect the finite polariton lifetime and assume that the polariton gas is at the thermal equilibrium. We account for the polariton-polariton repulsion. In analogy to many body spin problems we introduce macroscopic pseudospins descrbing the polariton condensates^54^. We note that a similar approach has been applied for the description of coherent phenomena in atomic gases^55^.

We shall use the pseudo-spin operator representation for the Hamiltonian describing our exciton-polariton JJ model system that has a form (the details of the model are presented in the Supplementary Material):

1 $$\hat{H}=\alpha {\hat{S}}_{z}^{2}+\beta {\hat{S}}_{x}^{2}-B{\hat{S}}_{x}.$$

**Figure 2:**
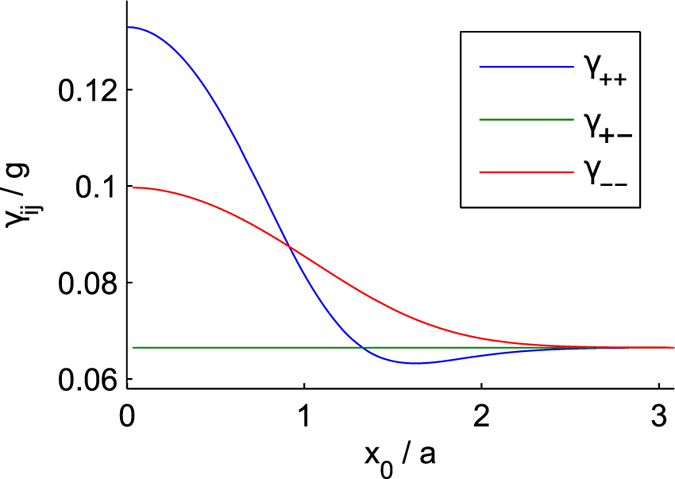
Dependences of the normalized matrix elements *γ*_*ij*_ *i*,*j* ∈ {+, −} on the normalized half inter-well distance *x*_0_/*a*; *a* is a characteristic size of the condensate wavefunction.

The coefficients in Eq. (1) characterize the tunnel coupling of two trapped condensates: the parameters *α* and *β* are proportional to the overlap integrals of real symmetric (Φ_+_) and anti-symmetric (Φ_−_) wavefunctions of two condensates that are ${\gamma }_{ij}=\int {{\rm{\Phi }}}_{i}^{2}{{\rm{\Phi }}}_{j}^{2}dx,i,j\in \{+,-\}$–see Fig. 2 and Suppl. Materials; *B* is determined by difference of chemical potentials which obey stationary Gross-Pitaevskii (GP) equations for two trapped condensates.

It is important to note that the last term in Eq. (1) characterizes the familiar XY–model Hamiltonian considered in refs 28, 29. The second term in (1) introduces an additional part that has no analogy in the XY-Hamiltonian; it is proportional to cos(2*ϕ*), where *ϕ* is the phase difference between two polariton condensates. This term vanishes in the limit ${x}_{0}/a\gg 1$, see Fig. 2 and Suppl. Materials^31, 33, 35^.

It is convenient to introduce the pseudo-spin operators in a form

2a $${\hat{S}}_{x}=s\,\cos \,\varphi -\,\sin \,\varphi \frac{d}{d\varphi },$$

2b $${\hat{S}}_{y}=s\,\sin \,\varphi +\,\cos \,\varphi \frac{d}{d\varphi },$$

2c $${\hat{S}}_{z}=-i\frac{d}{d\varphi },$$

The operators defined in Eq. (2) obey familiar *SU*(2) algebra commutation relations $[{\hat{S}}_{i},{\hat{S}}_{j}]=i{\varepsilon }_{ijk}{\hat{S}}_{k},i,j,k=x,y,z$. After some straightforward calculations from (1) one can obtain

3 $$\begin{array}{rcl}H & = & -(\alpha -\beta {\sin }^{2}\varphi )\frac{{d}^{2}}{d{\varphi }^{2}}-(\beta (s-\frac{1}{2})\sin \,2\varphi -B\,\sin \,\varphi )\frac{d}{d\varphi }\\  &  & -\,Bs\,\cos \,\varphi -\beta {s}^{2}{\sin }^{2}\varphi -\beta s{\cos }^{2}\varphi .\end{array}$$

Thereafter we assume that both condensates are composed by macroscopically large numbers of particles, i.e. the $s=N/2\gg 1$ is C - number. The general stationary Schrödinger equation with a Hamiltonian (3) writes:

4 $$\begin{array}{l}(\alpha -\beta {\sin }^{2}\varphi )\frac{{d}^{2}{\rm{\Phi }}}{d{\varphi }^{2}}+(\beta s\,\sin \,2\varphi -B\,\sin \,\varphi ))\frac{d{\rm{\Phi }}}{d\varphi }\\ \quad +\,(E+Bs\,\cos \,\phi +\beta {s}^{2}{\sin }^{2}\varphi ){\rm{\Phi }}=0,\end{array}$$

where Φ ≡ Φ(*ϕ*) is some 2*π*-periodic wavefunction that characterizes quantum phase properties^56^. It is possible to eliminate the term with first derivative in Eq. (4). In order to find the explicit form of the function Φ, it is convenient to substitute it by:

5 $${\rm{\Phi }}(\varphi )={\rm{\Psi }}(z)\,\exp (s\,\mathrm{ln}\,{\rm{dn}}z-\frac{{\rm{\Lambda }}s}{2\sqrt{\lambda \mathrm{(1}-\lambda )}}\arctan (\sqrt{\frac{\lambda }{1-\lambda }}{\rm{cn}}z)),$$

where $z={\int }_{0}^{\varphi }\frac{d\xi }{\sqrt{1-\lambda {\sin }^{2}\xi }}=F(\varphi ,{\rm{\lambda }})$ is a new phase variable; *F*(*ϕ*, λ) is the incomplete elliptic integral of the first kind. In (5) we have introduced dimension-less parameters ${\rm{\Lambda }}=\frac{B}{\alpha s}$, $\lambda =\frac{\beta }{\alpha }$. Inserting (5) into (4) we arrive to the familiar form of a Schrödinger equation

6 $$\alpha \frac{{d}^{2}{\rm{\Psi }}}{d{z}^{2}}+(E-V(z)){\rm{\Psi }}=0.$$

This equation describes an effective particle with the mass $m=\frac{{\hslash }^{2}}{2\alpha }$ and energy *E*, that is confined by the potential *V*(*z*) = *αs* ^2^ *V*_0_(*z*) with

7 $${V}_{0}(z)=\frac{(\frac{1}{4}{{\rm{\Lambda }}}^{2}-\lambda \mathrm{(1}-\lambda )){{\rm{sn}}}^{2}z-{\rm{\Lambda }}\mathrm{cn}z}{{{\rm{dn}}}^{2}z}\mathrm{.}$$

**Figure 3:**
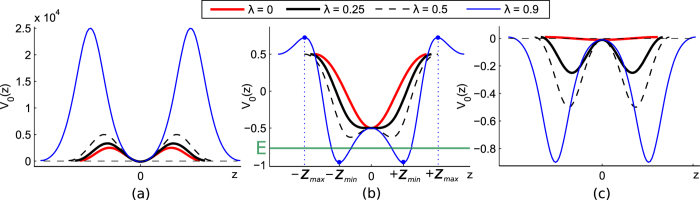
Effective dimension-less potential *V*_0_(*z*) for (**a**) Λ = 100, (**b**) Λ = 0.5, and (**c**) Λ = 0.01 as a function of the elliptic integral phase coordinate *z*. Points ±*z*_*min*_ and ±*z*_*max*_ in (**b**) correspond to global minima and maxima of the potential, respectively; *E* is the energy of the particle that experiences either quantum tunneling or thermal activation.

The dependences of the trapping potential *V*_0_(*z*) on the phase variable *z* are shown in Fig. 3. The period of the functions is 4*K*(*λ*). The dependence of the *λ*–parameter on normalized half of the inter-well distance $\frac{{x}_{0}}{a}$ can be approximated by $\lambda =0.5{(\exp [\frac{2{x}_{0}^{2}}{{a}^{2}}]-1)}^{-1}$ by using variational approach, see Suppl. Materials. The well-known behaviour of the quantum phase mesoscopic JJ (that map to the XY–model^28, 29^) can be recovered from (1), (7) at *λ* = 0 and shown by red curves in Fig. 3. This limit corresponds to infinitely large inter-well distances, with *x*_0_ → ∞, Fig. 2. On the other hand, the *λ*–parameter exhibits a sharp increase at $\frac{{x}_{0}}{a}\ll 1$. Obviously, in this limit our exciton polariton JJ model based on the assumption of a relatively weak coupling between trapped condensates breaks down. Below we are focusing on *λ*–parameters which belong to the range of 0 < *λ* < 1 and correspond to the moderate values of $\frac{{x}_{0}}{a}$ such as $\frac{{x}_{0}}{a}\ge 0.45$. The analysis of the quantum phase can be performed in three domains determined by vital Λ–parameter values^56^.

*Rabi regime* ${\rm{\Lambda }}\gg 1$. In this limit the trapping potential can be approximated by ${V}_{R}(z)=\frac{1}{4}{{\rm{\Lambda }}}^{2}{{\rm{sd}}}^{2}z$. Physically, for any value of *λ*–parameter belonging to the domain 0 < *λ* < 1 the system exhibits (Rabi) oscillations for the phase. In particular, small amplitude oscillations which are inherent to familiar Rabi regime can be achieved for negligable *λ*–see Fig. 3a and ref. 56.

*Fock regime* ${\rm{\Lambda }}\ll \mathrm{1/}{N}^{2}$. In purely quantum limit Φ(*ϕ*) → (2*π*)^−1/2^. In this regime, when inequality ${\rm{\Lambda }}\ll \lambda  < 1$ is held, Eq. (6) transforms to the Mathieu equation that is characterised by the set of eignen states consisting of all Mathieu functions.

*Josephson regime* $1/{N}^{2}\ll {\rm{\Lambda }} < 1$. This regime corresponds to the intermediate case between Rabi and Fock limits–see Fig. 3b. The behavior of the phase depends on the ratio between the parameters Λ and *λ*. If Λ ≥ 2*λ* the potential *V*_0_(*z*) possesses only one minimum at *z* = 0 and *V*(0) = −Λ.

In the opposite limit, for Λ < 2*λ*, the effective quantum “particle” described by Eq. (6) is trapped at the two minima of *V*_0_(*z*) that correspond to the *W*–like potential with coordinates ${z}_{min}=\pm {{\rm{cn}}}^{-1}\frac{{\rm{\Lambda }}}{2\lambda }$ and appearing for both of Josephson and Fock regimes, see Fig. 3b,c. The depth of the potential minima depends on the *λ* parameter. It is interesting to note that for Λ > 2(1 − *λ*) the potential *V*_0_(*z*) possesses a local minimum at ±2*K*(*λ*), see Fig. 1b.

## Annealing problem versus Quantum-classical PT’s

### Quantum-classical PT’s

Experimentally, exciton-polariton condensates are being observed at elevated temperatures, up to the room temperature in wide-gap semiconductor microcavities^1, 2^. At high temperatures the validity of the quantum coherent treatment of the Josephson problem is limited. In this section, we study the limits of validity of the quantum approach which also set the limits of the system suitability for quantum annealing^25^.

To be more specific we consider the tunneling in an effective *W*–like potential shown in Fig. 3b. We assume the thermal equilibrium condition to be fulfilled for an ensemble of exciton-polaritons at finite temperatures *T*, that would correspond to a hypothetic case of polaritons with the infinite life-time. We note in this connection that in the recent experiments^44^ the measured polariton lifetime was as long as 275 *ps*, which would be sufficient to justify the above approximation.

A transition between two stable states (say, between points −*z*_*min*_ and +*z*_*min*_ in Fig. 3b) can happen either through the quantum tunneling or, in classical way, due to the thermal activation. Below we show that the crossover between this two regimes may possess features of either a first (1st)- or second (2nd)-order phase transition. Obviously, at high temperatures such as *k*_*B*_ *T* ≥ Δ*V* (Δ*V* is the height of the barrier between two states with minimum of potential energy) the particle jumps over the barrier governed by the thermoactivation (Arrhenius) escape rate ${{\rm{\Gamma }}}_{T} \sim {e}^{-{\rm{\Delta }}V/{k}_{B}T}$ ^47^. This process is inherent to the classical (thermal) annealing problem^25^. In the “low temperature” limit ${k}_{B}T\ll {\rm{\Delta }}V$ polaritons undergo quantum tunneling through the barrier with a vanishing rate

8 $${\rm{\Gamma }} \sim {e}^{-{S}_{min}/\hslash },$$

where *S*_*min*_ is minimal value of the action, that excludes realisation of the quantum annealing scheme. At the crossover temperature *T*_*c*_ = *ħ*Δ*V*/*k*_*B*_ *S*_*min*_ the matching condition for the tunelling rates may be anticipated.

Our description is based on imaginary time path integral approach^50^. The imaginary-time action obtained within the WKB method approaches as

9 $$S(E)=\oint (\tfrac{1}{2}m{\dot{z}}^{2}+V(z))d\tau .$$

At the temperatures below the barrier height Δ*V* Eq. (9) enables one finding the escape rate Γ_*T*_^45^

10 $${{\rm{\Gamma }}}_{T} \sim {\int }_{0}^{{\rm{\Delta }}V}{e}^{-(E+{k}_{B}TS(E)/\hslash )/{k}_{B}T}\,dE={\int }_{0}^{{\rm{\Delta }}V}{e}^{-F/{k}_{B}T}dE,$$

where

11 $$F=E+{k}_{B}TS(E)/\hslash $$

plays role of the free energy of the system. The trajectories which minimize the action *S*_*T*_ obey the classical equation of motion $m\ddot{z}=\frac{dV}{dz}$ written for the thermon “particle” that oscillates within the inverted potential −*V*(*z*). Periodic solutions of this equation satisfy

12 $$\tfrac{1}{2}m{\dot{z}}^{2}=V(z)-E({\tau }_{p}),$$

where *τ*_*p*_ = *ħ*/*k*_*B*_ *T* is a thermon period corresponding to the energy *E*(*τ*_*p*_) and shown in Fig. 3b;

13 $${\tau }_{p}(E)=\sqrt{2m}{\int }_{{z}_{1}(E)}^{{z}_{2}(E)}\frac{dz}{\sqrt{V(z)-E}}.$$

In (13) the *z*_1,2_(*E*) are the turning points, see Fig. 3b. Equations (12,13) taken at the energy *E* = 0 and temperature *T* = 0 characterize the instanton solution with an infinite period. Remarkably in this case from (10) one can immediately obtain Eq. (8) that yields the escape rate in the quantum domain.

Combining (9) with (12) we arrive to

14 $${S}_{T}=2\sqrt{2m}{\int }_{{z}_{1}(E)}^{{z}_{2}(E)}\sqrt{V(z)-E}\,dz+E{\tau }_{p}(E).$$

To reveal the thermodynamic properties of the system it is necessary to consider small amplitude oscillations at the bottom, *z* = 0, of the inverted potential −*V*(*z*). The action in this case reads:

15 $${S}_{0}={\rm{\Delta }}V{\tau }_{p}(E).$$

The expansion of the potential *V*(*z*) into a series and excluding the constant value *V*(0) gives:

16 $$\begin{array}{rcl}V(z) & = & \alpha {s}^{2}({c}_{2}{z}^{2}+{c}_{4}{z}^{4}+o({z}^{4})),\end{array}$$

where *c*_2_ and *c*_4_ are coefficients defined as

17 $${c}_{2}=\frac{1}{4}{{\rm{\Lambda }}}^{2}-\frac{2\lambda -1}{2}{\rm{\Lambda }}-\lambda (1-\lambda ),$$

18 $${c}_{4}=\frac{2\lambda -1}{12}{{\rm{\Lambda }}}^{2}-\frac{16{\lambda }^{2}-16\lambda +1}{24}{\rm{\Lambda }}-\frac{\lambda (1-\lambda )(2\lambda -1)}{3}.$$

In particular, if the inverted potential −*V*(*z*) has the form *z* ^2^ − *z* ^4^ (*c*_2_ < 0, *c*_4_ > 0) the 2nd order phase transition occurs. If −*V*(*z*) behaves as *z* ^2^ + *z* ^4^ (*c*_2_ < 0, *c*_4_ < 0) the 1st order phase transition take place. The phase boundary between the 1st and 2nd order phase transitions is determined by the relation

19 $${\rm{\Lambda }}=\frac{1-16\lambda +16{\lambda }^{2}+\sqrt{1+32\lambda -32{\lambda }^{2}}}{\mathrm{4(2}\lambda -\mathrm{1)}}\mathrm{.}$$

**Figure 4:**
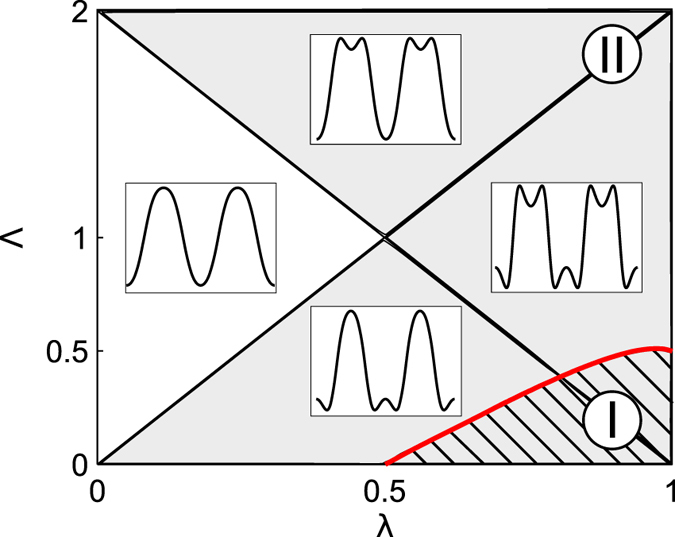
Diagram of the phase boundary for the 1st- and 2nd-order PT’s in Λ, *λ*–parameters plane. The PEL’s are shown in the windows.

We summarize our results in Fig. 4. An inset demonstrates various types of phase potentials *V*_0_(*z*) inherent to our exciton-polariton JJ model. The 1st order PT occurs for two type of potentials in the shaded region bounded by bold (red) curve. Second order PT’s appear for the potential landscapes taken from the dark area. The crossover temperature ${T}_{c}^{(2)}=\hslash {\omega }_{0}/2\pi {k}_{B}$ of 2nd-order PT transition is

20 $${T}_{c}^{(2)}={T}_{0}\sqrt{\lambda (1-\lambda )+(2\lambda -1)\tfrac{{\rm{\Lambda }}}{2}-\tfrac{1}{4}{{\rm{\Lambda }}}^{2}},$$

where ${\omega }_{0}=\frac{2\alpha s}{\hslash }\sqrt{\lambda \mathrm{(1}-\lambda )+\tfrac{1}{2}\mathrm{(2}\lambda -\mathrm{1)}{\rm{\Lambda }}-\tfrac{1}{4}{{\rm{\Lambda }}}^{2}}$ is a frequency of small oscillations of the thermon “particle” near the bottom of inverted potential, *T*_0_ = *αN*/2*πk*_*B*_ is a characteristic temperature that inherent to exciton polariton system. The *T*_0_ implies important time scale *τ*_0_ = 2*πħ*/*αN* that can be understood as thermon particle “lifetime”. Notably, as it is follows from (20) there is no barrier at *z* = 0 for Λ ≥ 2*λ*–see the white domain in Fig. 4.

**Figure 5:**
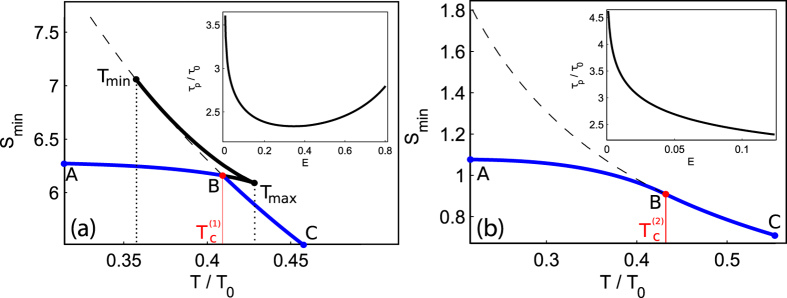
Dependences of the minimal action *S*_*min*_ (ABC-(blue) curve) taken in *sħ* units as a function of the normalized temperature *T*/*T*_0_ for (**a**) 1st-order PT, *λ* = 0.9, Λ = 0.1; and, (**b**) 2nd-order PT, *λ* = 0.5, Λ = 0.5; the solid (bold) line corresponds to the thermon action, *S*_*T*_; the dashed line corresponds to the thermodynamic action, *S*_0_ = *ħ*Δ*V*/*k*_*B*_ *T*, *S*_*min*_ = min{*S*_0_, *S*_*T*_}. The inset shows the dependences of normalized thermon period *τ*_*p*_/*τ*_0_ on energy *E* expressed in *αs* ^2^ units.

In Fig. 5a we represent the results exhibiting 1st-order PT inherent to narrow shadow domain in Fig. 4 where discontinuity of the derivative *dS*/*dT* occurs. It is clearly seen that the first derivative of *S*_*min*_ is discontinuous in this case and the dependence of the normalized thermon period *τ*_*p*_/*τ*_0_ on the energy *E* is non-monotonic, see the inset in Fig. 5a. The critical temperature ${T}_{c}^{\mathrm{(1)}}$ belongs to the temperature domain ${T}_{min} < {T}_{c}^{\mathrm{(1)}} < {T}_{max}$ and it can be found out numerically by solving Eqs (13–15) in the particular case of *S*_*T*_ = *S*_0_.

Analytically, the critical temperature ${T}_{c}^{\mathrm{(1)}}$ may be estimated from ${T}_{c}^{\mathrm{(1)}}=\hslash \Delta V/{k}_{B}{S}_{inst}$, where *S*_*inst*_ is an instanton action that can be represented as *S*_*inst*_ = *S*(*E*_*min*_). After some straightforward calculations for *S*_*inst*_ we obtain

21 $$\begin{array}{c}{S}_{inst}=S({E}_{min})=2s\hslash [\mathrm{ln}\,\frac{2\sqrt{\lambda }+\sqrt{4{\lambda }^{2}-{{\rm{\Lambda }}}^{2}}}{2\sqrt{\lambda }-\sqrt{4{\lambda }^{2}-{{\rm{\Lambda }}}^{2}}}-\frac{{\rm{\Lambda }}}{\sqrt{\lambda \mathrm{(1}-\lambda )}}\arctan (\frac{\sqrt{\mathrm{(1}-\lambda \mathrm{)(4}{\lambda }^{2}-{{\rm{\Lambda }}}^{2})}}{{\rm{\Lambda }}})]\mathrm{.}\end{array}$$

Figure 5b displays 2nd-order PT from quantum (solid bold line of *S*_*T*_) to thermal (classical) regimes–dashed bold line of *S*_0_. The crossover occurs at the critical temperature ${T}_{c}^{\mathrm{(2)}}$ where *S*_*T*_ = *S*_0_ and *E* = *E*_0_. The inset demonstrates a monotonic dependence of the normalized thermon period *τ*_*p*_/*τ*_0_ on energy *E* that is inherent to second-order phase transition. Closest to the critical point with energy *E* = *E*_0_ the thermon undergoes small amplitude oscillations.

Remarkably, form Fig. 5 it is clearly seen that at the temperatures sufficiently below the critical temperatures *T*_*c*_ the action *S*_*min*_ is temperature independent that corresponds to the quantum regime of macroscopic tunneling. The connection with phenomenological Landau theory of PT’s^57^ can be obtained by introducing the “order parameter” *P* that is^45^

22 $$P=\sqrt{\frac{V(0)-E}{{\rm{\Delta }}V}}.$$

Taking into account Eq. (11) for normalized free energy *F*(*P*) one can obtain

23 $$\frac{F(P)}{{\rm{\Delta }}V}=1+(\theta -1){P}^{2}+\theta \eta {P}^{4}+O({P}^{6}),$$

where $\eta =3{c}_{4}{({\rm{\Lambda }}-2\lambda )}^{2}/32{c}_{2}^{2}\lambda$ and $\theta =T/{T}_{c}^{(2)}$ is normalised temperature parameter. It is important that sign of second term in Eq. (23) is completely determined by sign of *c*_4_ -coefficient, see (18), that implies whether 1st-, or 2nd-order PT occurs in the system.

**Figure 6:**
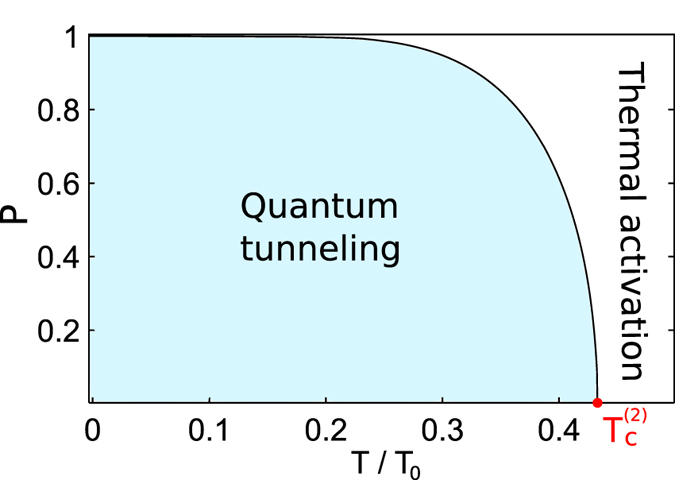
The dependence of the *P*–parameter on the normalized temperature *T*/*T*_0_. The parameters *λ*, Λ are the same as in Fig. 5b.

Thus, *P*–parameter describes features of potential shape for the “particle” possessing energy *E* and characterizing PT in the system according to Landau theory^57^–see Fig. 3b. Figure 6 shows the behaviour of the introduced order parameter. One can see that the phases corresponding to the quantum tunneling and classical thermal activations are separated by the second order phase transition.

**Figure 7:**
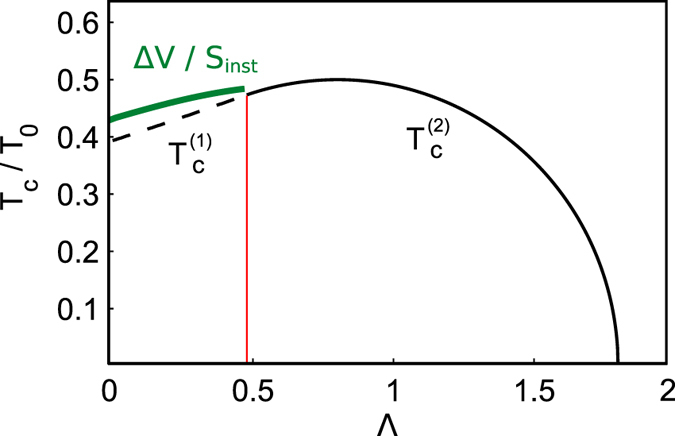
The dependence of the normalized critical temperature *T*_*c*_/*T*_0_ as a function of the parameter Λ. The bold (green) line corresponds to the analytical expression for ${T}_{c}^{(1)}$ given by Eq. (21).

In the optical experiments one usually has a better control of the exciton-polariton density than their effective temperature, see e.g. refs 1, 2. For instance, the density of polariton gas can be changed by varying the optical pump intensity. In Fig. 7 we plot a numerically calculated critical temperature of the 1st and 2nd order PTs as a function of the Λ-parameter for experimentally accessible semiconductor JJ samples. The bold (green) line corresponds to the analytical solution obtained with Eq. (21). Since the Λ-parameter is inversely proportional to the density of the exciton-polariton gas (and the blue-shift of the energy of the corresponding photoluminescence peak) Fig. 7 establishes an important relation between the critical temperatures discussed in the paper and the relevant exciton polariton densities.

Now let us consider the decay of the state of the system that corresponds to the upper minimum of the potential *V*(*z*) located at ±2*K*(*λ*) in Fig. 3b and exists for Λ > 2(1 − *λ*). Physically, the decay takes place because of the tunneling of our effective particle through the barrier located at one of the points ${z}_{max}=\pm {{\rm{cn}}}^{-1}(-\frac{\mathrm{2(1}-\lambda )}{{\rm{\Lambda }}})$. By expanding the potential *V*(*z*) in the vicinity of *z*_*max*_ it is possible to show that only the 2nd-order PT with a temperature ${T}_{c}^{\mathrm{(2)}}=({T}_{0}\mathrm{/2)}\sqrt{\frac{{{\rm{\Lambda }}}^{2}}{1-\lambda }-\mathrm{4(1}-\lambda )}$ may take place in this case.

### Annealing

Nowadays simulated annealing (SA) algorithm is at the heart of the optimization problem in computer science, see e.g. ref. 58. In particular, it enables to solve so-called *NP*-hard optimization problems that is met in our life frequently; an example, we refer here to the traveling salesman problem, see e.g. ref. 59. From the point of view of the physical implementation, the optimization algorithm implies searching of a global minimum for PEL that relays to *N* particle spin system oriented randomly^25^. According to the SA approach the minimum can be achieved by physical system as a result of the deep cooling. Apart from SA, quantum annealing (QA) uses the quantum tunneling option to achieve a global minimum. In some aspects QA algorithm is relevant to the so-called adiabatic quantum computing scheme^60^. Although in this paper we are not aiming at detail investigation of various annealing schemes, some of their features become evident from our previous analysis.

Actually, at high enough temperatures, the system may visit the higher-energy eigen states by means of the thermal activation and due to thermal fluctuations, see Fig. 1. Certainly, at zero temperature, being at the full thermodynamic equilibrium state, one can find the configuration of the spin system at PEL minimum possessing also minimum of it free energy *F* and action *S*_*min*_, respectively, see Eqs (11,23). Clearly, the last one corresponds to the implementation of QA algorithm when system undergoes quantum tunneling instead of thermal activation. The transition between this two scenario, in fact, reflects the nature of SA scheme that lies in the crossover from the thermal (classical) to the quantum annealing regime.

From the practical point of view, it is instructive to estimate the time that is required for the system to perform the computation. Obviously this time is limited by characteristic tunneling time, or by the characteristic hopping time. Here we estimate this time simply as

24 $${t}_{T}=\hslash /{{\rm{\Gamma }}}_{T}.$$

Obviously, the characteristic time scale *t*_*T*_ depends on the temperature of the system. At *T* = 0, where only the QA is possible, from (24) we obtain

25 $${t}_{0}={e}^{{S}_{min}/\hslash }\mathrm{.}$$

One can see that the parameter *t*_0_ defines limiting (minimal) time required to perform QA for our toy optimization model. Notably, this time depends on the number of particles as *t*_0_ = *e* ^*Nγ*′^, where *γ*′ is some constant. Since relation *S*_*T*_ > *S*_*min*_ is still valid up to the temperatures of PT we have *t*_*T*_ > *t*_0_ in the same limit. Thus, from (24) and (25) immediately follows that improvement of the annealing algorithm in the quantum domain strongly depends on the particle number *N* and governed by the ratio ${t}_{T}/{t}_{0}={e}^{({S}_{T}-{S}_{min})/\hslash }$ which depends on the effective action difference taken at a given temperature *T*; this difference clearly seen from Fig. 5a for the 1st order PT problem where thermal and quantum regimes coexist.

Slightly above the critical temperature *T*_*c*_ the thermal (classical) SA algorithm is realised in our system. At relatively high temperatures it does not guarantee the system relaxation to the global minimum. Moreover, some non-equilibrium processes can occur in this case and the model used here may be no more valid (see next Section). The predictions of the present model must be valid in the vicinity of critical point and below.

Let us briefly discuss methods of the experimental study of the quantum-classical PT for the polariton JJ’s. Our estimations show that, for the experimentally accessible polariton interaction strength of *αN* = 0.6 *meV*, characteristic of narrow-band semiconductor samples, *T*_0_ is about 1.1 *K* that corresponds to the thermon effective lifetime of *τ*_0_ ≈ 7 *ps*. The value of *T*_0_ is comparable with a typical temperature of a BEC, or, with temperature of the Berezinsky-Kosterlitz-Thouless PT predicted for the dilute weakly interacting exciton-polariton gas at thermal equilibrium^1, 2^. Some further enhancement of *T*_0_ may be achieved by the increase of the optical pumping intensity and varying the detuning of exciton and photon modes in a microcavity^44^.

## PT’s in the presence of dissipation

Now let us consider the exciton polariton JJ accounting for the finite exciton polariton lifetime *τ*_*pol*_. The finite radiative lifetime is a important characteristic of any polariton system and, strictly speaking, can never be ignored. Obviously, in order to allow for the Josephson oscillations, the characteristic time *τ*_0_ that is responsible for quantum tunneling effects should be shorter than all characteristic times describing non-equilibrium processes in the exciton-polariton gas, including the radiative decay. Hence,

26 $${\tau }_{0}\ll {\tau }_{pol}.$$

The condition (26) is fulfilled in the experimental work^44^, in particular. Here we examine a different situation namely, the case where the exciton polariton lifetime *τ*_*pol*_ is comparable with *τ*_0_. Note that this situation is realized the multiple experiments dealing with non-equilibrium exciton polariton condensates, see e.g. refs 1, 2, 16–18.

To be more specific in what follows we shall consider the influence of the dissipation on the exciton polariton JJ quantum phase properties in the adiabatic limit^61^. We start from GP equations obtained from (see Supplementary equation (S5)) for the mean fields ${\psi }_{\mathrm{1,2}}=\langle {\hat{\psi }}_{\mathrm{1,2}}\rangle$ at Γ = 0 (*B* = *G*), which read as

27 $$i\hslash {\dot{\psi }}_{1,2}=-i\kappa {\psi }_{1,2}+(A{|{\psi }_{1,2}|}^{2}+2C{|{\psi }_{2,1}|}^{2}){\psi }_{1,2}-(B/2-C{\psi }_{1,2}^{\ast }{\psi }_{2,1}){\psi }_{2,1}.$$

In (27) we have introduced the dissipation term with $\kappa \simeq \hslash /{\tau }_{pol}$. Defining new variables Ψ_1,2_ as *ψ*_1,2_ = Ψ_1,2_exp(−*κt*/*ħ*) for the mean field pseudo-spin components (see Supplementary equation (S6)). We obtain from (27)

28 $$\begin{array}{rcl}{\dot{S}}_{x} & = & -2\alpha ^{\prime} (t){S}_{z}{S}_{y};\\ {\dot{S}}_{y} & = & 2(\alpha ^{\prime} (t)-\beta ^{\prime} (t)){S}_{z}{S}_{x}+{S}_{z}.\\ {\dot{S}}_{z} & = & 2\beta ^{\prime} (t){S}_{x}{S}_{y}-{S}_{y};\end{array}$$

Here we have introduced the dimensionless time *t*′ = *tB*/*ħ*.

Set of Eq. (28) describes the dynamics of normalized mean field pseudo-spin parameters on the Bloch sphere with ${S}_{x}^{2}+{S}_{y}^{2}+{S}_{z}^{2}=1$. Equation (28) look similar to those written for a non-dissipative system but with the time dependent parameters *α*′(*t*) = (1/Λ)exp(−2*κ*′*t*′), *β*′(*t*) = (*λ*/Λ)exp(−2*κ*′*t*′), *κ*′ = *κ*/*B* ^62^. Taken out the dissipation, Eq. (28) possesses a bifurcation point *λ*/Λ = 1/2 that corresponds to the solid black curve in Fig. 3b.

**Figure 8:**
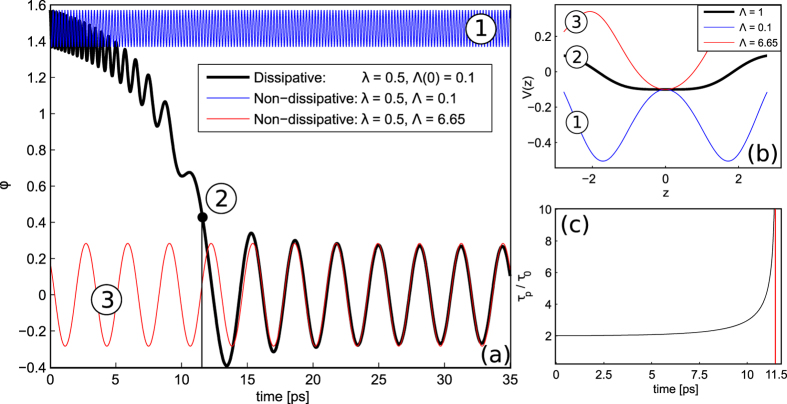
(**a**) Normalized phase difference as a function of time; (**b**) effective potential vs phase parameter *z* for red, black and blue curves, respectively, and (**c**) reduced thermon period *τ*_*p*_/*τ*_0_ vs time for *κ*/*ħ* = 0.1 *THz*. Initial conditions are *S*_*z*_(0) = *S*_*x*_(0) = 0, *S*_*y*_(0) = 1, and *ϕ*(0) = *π*/2, respectively.

In Fig. 8a we plot the phase difference between the condensates *ϕ* = arctan(−*S*_*y*_/*S*_*x*_) as a function on time. A non-dissipative system has two different regimes depending on the values of the *β*–parameter, that are blue (labeled as 1) and red (labeled as 3) curves in Fig. 8, respectively. The blue curve is plotted for the case of *β*′ > 1/2 and it corresponds to the quantum regime–see Fig. 8b. Contrary, the red curve describes the phase between two condensates below the threshold (*β*′ < 1/2) that corresponds to the absence of the barrier.

In the presence of dissipation (solid black curve in Fig. 8a) the phase of exciton-polariton JJ starts from one of the potential minima. We assume here that the temperature of the system is not sufficient for thermal activation and the quantum tunneling is possible. The system evolves adiabatically and then crosses the critical value *β* = 1/2 due to the decrease of the total number of particles caused by the radiative decay. The final (temporal) state represents the Josephson or, Rabi oscillation regime.

Figure 8b and c demonstrate the suppression of *W*–like potential and the relevant enhancement of the reduced thermon period *τ*_*p*_/*τ*_0_ which occurs at switching time *t*_*sw*_ = 11.5*ps* for ${\tau }_{pol} \sim \hslash /\kappa =10ps$, respectively.

Thus, behavior of the phase in the presence of dissipation showed in Fig. 8 is characteristic of a established non-equilibrium PT from the regime where tunneling is possible to the regime where tunneling is suppressed. However, this regime found at *t* > *t*_*sw*_ cannot be interpreted immediately as the classical one. Actually, the adiabaticity condition reads^61^:

29 $$\frac{1}{{\omega }^{2}(t)}|\frac{d\omega }{dt}|\ll 1.$$

where $\omega (t)=B\sqrt{(1-2\beta ^{\prime} (t))(1-2\beta ^{\prime} (t)+2\alpha ^{\prime} (t))}/\hslash$ is the frequency of small oscillations slowly depending on time^62^. The solution for the phase in this case can be approximated by

30 $$\varphi (\tau )\approx \frac{A}{\omega (\tau )}\,\cos (\omega (\tau )\tau ),$$

where *τ* is the time elapsed after the oscillation sets in, and *A* is the amplitude of oscillations at *τ* = 0. At large enough times, *ω*(*t*) ≈ *B*/*ħ* and permanent Rabi oscillations for the JJ phase *ϕ* are established, see Fig. 8a and ref. 63. To distinguish between the quantum and classical character of these oscillations, a purely quantum approache to the problem should be considered. It will be in the scope of our further research.

## Conclusion

We have studied theoretically the coupling of two spatially separated trapped exciton-polariton condensates at finite temperatures and accounting for the dissipation. We demonstrate the crossover from thermal to quantum annealing regime for a model system of two condensates localised by a W-shape potential. Second regime is classical one and characterizes thermal activation. The transition between these two regimes exhibits universal features of the 1st or 2nd order PTs which can be interpreted as PT between classical and quantum regimes. It is important that critical temperature of transition depends on some characteristic temperature parameter *T*_0_ (see e.g. (20)) that that is governed by the polariton-polariton interaction length (so-called blue shift) *αN*. It is expected that at the temperatures sufficiently higher than *T*_0_ the exciton-polariton JJ device operates in classical way. In this case the simulated thermal annealing algorithm might be implemented.

The influence of dissipation effects originating from the radiative decay of polaritons by photon tunneling through the Bragg mirrors is revealed by our analysis. The observation of quantum tunneling processes only becomes possible within short time intervals where the dissipation cannot essentially affected to the system. Otherwise, after some time interval the dissipation leads to crossover in the phase dynamics and it damages the W–potential as a whole, see Fig. 8. In this case permanent Rabi oscillations of JJ phase occurs within the adiabatic approach. To reveal either quantum of classical nature of these oscillations it is necessary to study the fluctuations of the exciton-polariton system beyond the semiclassical approach. For moderate dissipation rates and at temperatures (or relevant polariton gas densities) sufficiently below the temperature of *T*_0_ the exciton-polariton JJ device is suitable for various applications where the quantum tunneling effect is crucial.

We note that the quantum tunneling effect may be used for designing exciton polariton phase qubits similar to superconductor devices, but in the optical wavelength domain^52^. Actually, two minima located at the points −*z*_*min*_ and +*z*_*max*_ of *W*-shape quantum phase potential in Fig. 3b can be used for setting the exciton polariton qubit states | 0> and | 1> similarly to superconductor flux qubits. However, in our case such qubits may be tailored by the external optical or electrical pump. As a result, QA problem can be experimentally solved with use of the polariton-based qubits^64^.

## Electronic supplementary material

supplementary

LaTeX Supplementary File
