# Supplementary material for: Exciton-polariton Josephson junctions at finite temperatures
Source: Sci Rep. 2017 Aug 25;7:9515. doi: 10.1038/s41598-017-09824-8 (PMC5572052; doi:10.1038/s41598-017-09824-8)
Supplement: Supplementary file 1 — supplementary [file 41598_2017_9824_MOESM1_ESM.pdf]

Supplementary materials: Exciton-polariton Josephson junctions at finite temperatures

M. E. Lebedev[1],
[1], D. A. Dolinina[1], Kuo-Bin Hong[2], Tien-Chang Lu[2], A. V. Kavokin[3, 4, 5], and A. P. Alodjants[1, 6, *]

[1]ITMO University, St. Petersburg 197101, Russia
[2]Department of Photonics, National Chiao Tung University, Hsinchu 300, Taiwan
[3]Spin Optics Laboratory, St. Petersburg State University, Ulanovskaya, Peterhof, St. Petersburg 198504, Russia
[4]School of Physics and Astronomy, University of Southampton, SO17 1BJ Southampton, United Kingdom
[5]Istituto CNR-SPIN, Viale del Politecnico 1, I-00133, Rome, Italy
[6]Vladimir State University named after A. G. and N. G. Stoletovs, Gorkii Street 87, Vladimir, Russia
[*]alexander_ap@list.ru

To derive Eq.(1) we consider an equilibrium exciton polariton condensate trapped in a symmetric one dimensional double-well potential $U(x) = h((x/x_0)^2 - 1)^2$. Here $2x_0$ is the distance between potential minima, h is the depth of the potential. The Hamiltonian for the system of weakly interacting exciton polaritons trapped in an external potential $U(x)$ can be written in the using the secondary quanttization formalism as:

$$\hat{H} = \int \hat{\psi}^\dagger \Big\{ -\frac{\hbar^2}{2m_{pol}} \frac{d^2}{dx^2} + U(x) + \frac{g}{2} \hat{\psi}^\dagger \hat{\psi} \Big\} \hat{\psi} dx, \tag{S1}$$

where $\hat{\psi} \equiv \hat{\psi}(x,t)$ is a polariton field operator that annihilates a particle at the position x and time t, g describes the two-body (polariton-polariton) scattering length, m_{pol} is low branch polariton effective mass. In the paper we use so-called two-mode representation of $\hat{\psi}$ - operator:

$$\hat{\psi} = \hat{\psi}_1(t)\Phi_1(x) + \hat{\psi}_2(t)\Phi_2(x), \tag{S2}$$

where $\hat{\psi}_{1,2}$ are time dependent operators characterizing the condensates at the left and at the right wells respectively; $\Phi_{1,2}(x)$ are condensate wavefunctions in real space. It's convenient to represent $\Phi_{1,2}(x)$ as

$$\Phi_{1,2} = \frac{\Phi_+ \pm \Phi_-}{\sqrt{2}} \tag{S3}$$

where $\Phi_\pm(x) = \pm\Phi_\pm(-x)$ are symmetric (Φ_+) and antisymmetric (Φ_-) real wavefunctions obeying the stationary Gross-Pitaevskii equations

$$\mu_\pm \Phi_\pm = -\frac{\hbar^2}{2m_{pol}}\frac{d^2\Phi_\pm}{dx^2} + U(x)\Phi_\pm + g\Phi_\pm^3 \tag{S4}$$

and the normalization condition $\int \Phi_\pm^2 dx = 1$; $\mu_\pm$ is a chemical potential. As a result, an operator $N = \hat{\psi}_1{}^\dagger \hat{\psi}_1 + \hat{\psi}_2{}^\dagger \hat{\psi}_2$ characterizes the total number of particles. Substituting (S3), (S2) into (S1) we arrive to

$$\begin{aligned} \hat{H} \;=\; & \frac{A}{2}(\hat{\psi}_1^{\dagger 2}\hat{\psi}_1^2 + \hat{\psi}_2^{\dagger 2}\hat{\psi}_2^2) - \frac{G}{2}(\hat{\psi}_1^\dagger\hat{\psi}_2 + \hat{\psi}_1\hat{\psi}_2^\dagger) \\ & -\frac{\Gamma}{2}(\hat{\psi}_1^{\dagger 2}\hat{\psi}_1\hat{\psi}_2 + \hat{\psi}_1^\dagger\hat{\psi}_1^2\hat{\psi}_2^\dagger + \hat{\psi}_1^\dagger\hat{\psi}_2^\dagger\hat{\psi}_2^2 + \hat{\psi}_1\hat{\psi}_2^{\dagger 2}\hat{\psi}_2) \\ & +\frac{C}{2}(\hat{\psi}_1^{\dagger 2}\hat{\psi}_2^2 + 4\hat{\psi}_1^\dagger\hat{\psi}_1\hat{\psi}_2^\dagger\hat{\psi}_2 + \hat{\psi}_1^2\hat{\psi}_2^{\dagger 2}), \end{aligned} \tag{S5}$$

where we have denoted $\gamma_{ij} = g\int \Phi_i^2\Phi_j^2 dx, i,j \in \{+,-\}$, $A = \frac{1}{4}(\gamma_{++} + \gamma_{--} + 6\gamma_{+-})$, $\Gamma = \frac{1}{2}(\gamma_{--} - \gamma_{++})$, $C = \frac{1}{4}(\gamma_{++} + \gamma_{--} - 2\gamma_{+-})$, $G = \mu_- - \mu_+ - 2\Gamma$.

Hamiltonian (S5) describes the non-linear model of a Josephson junction effect with exciton polariton condensates trapped in a double-well potential. The terms containing Γ and C in (S5) characterize the non-linear contribitions to the tunneling of single polaritons and polariton pairs, respectively. The effective (nonlinear) tunneling rate governed by these terms is $G_{eff} = \frac{1}{2}(G + \Gamma N - C\hat{\psi}_1^\dagger\hat{\psi}_2)$.

The relevant coefficients can be estimated using the variational ansatz $\Phi_\pm = A_\pm\Big[\exp\Big(-\frac{(x-x_0)^2}{2a^2}\Big) \pm \exp\Big(-\frac{(x+x_0)^2}{2a^2}\Big)\Big]$, where a is the localisation length of the condensate wavefunctions in each of two potential wells which are assumed identical. The amplitudes A_+ and A_- are given by the normalization conditions. It is important that differences between various rates γ_{ij} rapidly vanish with the increasing the inter-well distance $2x_0$. We shall assume $\Gamma = C = 0$ for $x_0 >> a$. This limit corresponds to the familiar problem of two weakly linked condensates at zero temperature, see e.g. [1, 2, 3, 4]. Now let us introduce the pseudo spin operators

$$\hat{S}_x = \frac{1}{2}(\hat{\psi}_1^\dagger\hat{\psi}_2 + \hat{\psi}_1\hat{\psi}_2^\dagger) \tag{S6a}$$

$$\hat{S}_y = \frac{i}{2}(\hat{\psi}_1^\dagger\hat{\psi}_2 - \hat{\psi}_1\hat{\psi}_2^\dagger) \tag{S6b}$$

$$\hat{S}_z = \frac{1}{2}(\hat{\psi}_2^\dagger\hat{\psi}_2 - \hat{\psi}_1^\dagger\hat{\psi}_1). \tag{S6c}$$

Inserting (S6) into the (S5) we obtain Eq.(1) with $\alpha = A - C = 2\gamma_{\pm} > 0$, $\beta = 2C > 0$ and $B = \Gamma N + G > 0$. We also chose x_0 obeying the condition $\gamma_{++} \simeq \gamma_{--} = \gamma$ assuming $\Gamma = 0$ for simplicity.

References

[1] Aleiner, I. L., Altshuler, B. L. and Rubo, Yu. G., Radiative coupling and weak lasing of exciton-polariton condensates. *Phys. Rev. B*, **85** (2012).

[2] Shelykh, I. A., Solnyshkov, D. D., Pavlovic, G. and Malpuech, G., Josephson effects in condensates of excitons and exciton polaritons. *Phys. Rev. B*, **78** (2008).

[3] Borgh, M. O., Keeling, J. and Berloff, N. G., Spatial pattern formation and polarization dynamics of a nonequilibrium spinor polariton condensate. *Phys. Rev. B*, **81** (2010).

[4] Raghavan, S., Smerzi, A., Fantoni, S. and Shenoy, S. R., Coherent oscillations between two weakly coupled Bose-Einstein condensates: Josephson effects, π oscillations, and macroscopic quantum self-trapping. *Phys. Rev. A*, **59**, (1999).
